# Enhance or inhibit? Unveiling the influence of chairman’s hometown attachment on the corporate philanthropy–Corporate financial performance relationship

**DOI:** 10.3389/fpsyg.2022.956689

**Published:** 2022-11-09

**Authors:** Limin Chen, Xuelin Bu

**Affiliations:** ^1^School of Economics and Management, Wuhan University, Wuhan, China; ^2^Global Strategy Research Center at Wuhan University, Wuhan, China

**Keywords:** corporate philanthropy, corporate financial performance, hometown attachment, institutional legitimacy, Chinese context, culture

## Abstract

Although there have been numerous studies on the relationship between corporate philanthropy and corporate financial performance (CFP), theoretical analysis focusing on the legitimacy-based mechanism and the moderating role of key executives’ psychological characteristics is scarce. Hometown attachment is a special form of place attachment in environmental psychology, which reflects people’s psychological attachment to their hometown and the state of maintaining an intimate emotional connection with it. Based on Scott’s three-pillar institutional perspective, this study traces its origins from the Chinese cultural context, and discusses the legitimacy basis of donations in the Chinese cultural context and why donations can improve CFP. On this basis, the moderating effect of the chairman’s hometown attachment on the corporate donation–performance relationship is empirically tested. Through logical deduction, hometown attachment may form legitimacy pressure to weaken the above relationship or leverage more legitimacy resources to strengthen such a relationship. Which effect dominates? The empirical results in this study of A-share listed firms from 2009 to 2018 show that the moderating role of hometown attachment is more in line with the resource theory than the former pressure theory. Further research shows that the government and consumers are important providers of these legitimacy resources. This study reveals the mechanism for legitimacy acquisition through corporate donations in the Chinese context and answers the question of what the chairman’s hometown attachment brings about to their donation performance, providing some inspiration for practice.

## Introduction

How does corporate social responsibility (CSR), represented by corporate philanthropy, affect corporate financial performance (CFP)? Existing conclusions are diverse and even contradictory (Grewatsch and Kleindienst, 2017; Awaysheh et al., 2020; Wang et al., 2022). In addition to the dominant positive correlation in recent years (Godfrey, 2005; Wang and Qian, 2011), arguments for negative correlation (Friedman, 1970; Tian and Wang, 2017), uncorrelation (Surroca et al., 2010), U-shaped relationship (Brammer and Millington, 2008; Barnett and Salomon, 2012) and inverted U-shaped relationship (Wang et al., 2008) are emerging. Grewatsch and Kleindienst (2017) joked that, regarding the problem of social responsibility and CFP, “we as researchers are able to argue and find whatever shape we want the … relationship to have” (p. 384). Inconsistent conclusions have sparked enthusiasm in research among scholars. More start to study “when” instead of “whether” social responsibility boosts CFP and encourage scenario-based studies to provide a “finer-grained and differentiated picture.” Although research on the relationship between corporate philanthropy and CFP has been done based on the stakeholder theory, resource-based view, risk management, signaling theory, and agency theory (e.g., Porter and Kramer, 2002; Godfrey, 2005; Wang and Qian, 2011; Tian and Wang, 2017; Flammer, 2018; Surroca et al., 2020), the investigation from the perspective of institutional legitimacy mainly focuses on explaining the motivations of social responsibility or the strategies of multinational firms. Given the very different institutional origins of Chinese and Western societies, this study argues that it is necessary to examine corporate philanthropy, the oldest and most prevalent form of organizational prosocial behavior, in the unique Chinese cultural context to clarify its social role and impact on corporate legitimacy. Therefore, the first objective of this study is to explore how Chinese firms enhance their performance through corporate philanthropy from the perspective of legitimacy foundations and the role of donations in a specific Chinese cultural context.

The reason why we select the perspective of legitimacy as the theoretical foundation is out of two reasons. Practically, CSR is a kind of typical behavior to obtain social legitimacy and even improve corporate performance. In July 2021, waterlogging and flooding caused by sudden torrential rains in many parts of northern Henan Province aroused nationwide attention. Hongxing Erke, a private enterprise, donated RMB50 million to the disaster-hit areas immediately, which was praised and publicized by many netizens spontaneously. Customers poured into their online sales channels to buy its goods, raising the sales volume to top RMB100 million in 2 days^1^ accessed date [2022-1-10]. The case vividly demonstrates “those who benefit others also benefit themselves.” Corporate philanthropy as a redistribution of wealth is valued by China’s leadership and has aroused attention among policymakers.

Theoretically, the survival and development of an organization in a given society requires necessary legitimacy based on an institutional perspective (Meyer and Rowan, 1977). Suchman (1995, p. 574) suggested that “legitimacy is a generalized perception or assumption that the actions of an entity are desirable, proper or appropriate within some socially constructed system of norms, values, beliefs, and definitions.” Firms undertaking social responsibility is in line with the public interests and expectations, which can gain themselves certain legitimacy. Meanwhile, the public’s values and norms in turn determine the specific scope, content, goals, and implementation of the CSR. Several studies have shown that CSR commitment highly depends on social institutions and perceptions (Campbell, 2007; Matten and Moon, 2020). However, the existing research has only explored the impact of the institutional context on CSR behavior or commitment but did not further reveal how the legitimacy context provided by the given society in which the firm operates constrains the performance of corporate philanthropy. Approaching from the angle of legitimacy, however, can help fill this research gap.

Hometown attachment originates from a concept in environmental psychology and is a special form of place attachment. The definition of place attachment is not unanimous and varies with the research focus and differences in disciplines (Scannell and Gifford, 2010). According to the definition proposed by Hidalgo and HernÁndez (2001, p. 274), place attachment is “a positive, affective bond between an individual and a specific place, the main characteristic of which is to maintain closeness to such a place.” Therefore, this study defines hometown attachment as the positive emotions such as attachment, love, pride, and identification with one’s hometown and the state of maintaining an intimate emotional connection with one’s hometown. According to a relevant study in environmental psychology, this emotional connection can be manifested at both the individual and organizational levels: Individual place attachment is based on a stable sense of self constructed relying on one’s experiences in and memories of a particular place, while at the organizational level, it is based on the culture, perceptions, and experiences shared by members in a specific place (Scannell and Gifford, 2010). So, hometown attachment can be seen as a stable and continuous individual psychological characteristic. The Chinese people are deeply attached to their homeland. Overseas Chinese often travel thousands of miles back to seek their roots. Idioms such as “Return to one’s hometown with honor,” “Displaced from one’s hometown,” and “Fallen leaves return to the roots” all reflect the solid attachment to the hometown of the Chinese nation. However, internationally, academic research on place attachment is mainly in the field of environmental psychology and the research on hometown attachment is just in its infancy. In Chinese academia, the studies about hometown attachment are slightly more, but mostly focused on the context of the agency problem. For instance, from the view of regional development, scholars suggested that officials are inclined to allocate more valuable resources to their hometown, resulting in a higher rate of economic development in their hometown than in other regions in the same period (Xu and Li, 2019). From the view of corporate governance, the fellowship relationship between CEOs and directors can increase corporate risks and violations (Lu and Hu, 2014). Besides, scholars found that when investing offsite, CEOs demonstrate a strong “hometown preference” for agency reasons rather than information advantage or familiarity (Cao et al., 2018). In CSR-related studies, Hu et al. (2017) used hometown attachment to explain the motivation of some Chinese firms to fulfill their environmental responsibilities actively. Because of hometown attachment, executives are more concerned about the environment in their hometown and their self-interest in sacrificing the environment for development is weakened. The hometown attachment also reduces conflicts between environmental stakeholders and corporate management and complements the local formal system to guide entrepreneurs to comply with environmental regulations. As an implicit psychological state, hometown attachment is not easy to be accurately identified and quantified. Questionnaire surveys are often used to collect this data in psychological research. However, the questionnaire survey has great limitations in the empirical test. Hu et al. (2017) innovatively adopted the “consistency of the chairman’s place of origin or birth and the business domicile” to identify whether the managers have a characteristic of hometown attachment, which has been widely accepted by relevant studies and paved the way for our research.

In addition, extant studies based on the upper echelon theory have focused on the association of CEO psychology with CSR engagement from different perspectives, such as narcissism and cognitive style (Li et al., 2020), and the influence of psychological factors embedded in life experiences, gender, and cultural background (Du et al., 2014; Petrenko et al., 2016; Galbreath, 2018; Xu and Ma, 2021; Zhao et al., 2022). However, the mechanism of how hometown attachment affects the performance of corporate donation is under-revealed. Since hometown attachment is one of the key psychological characteristics of executive, which influences not only entrepreneurs’ strategic behavior but also the outcomes of the strategy, does the chairman’s hometown attachment promote or weaken the relationship between donation and performance?

Therefore, the second objective of this study is to analyze two possible competing moderating roles of hometown attachment based on clarifying the mechanism of donation legitimacy and then determine which role dominates through an empirical test.

This study may make a marginal contribution to existing research in the following areas. First, this study enriches literature the corporate philanthropy–CFP relation by revealing the mechanism of legitimacy acquisition and resource transformation by donation in Chinese cultural context. Second, it shed light on the social role of the psychological phenomenon of entrepreneurs’ hometown attachment and promote the study and application of the psychological characteristics of key executives in the field of management.

## Literature review

The value creation effect of CSR represented by corporate philanthropy has long been controversial theoretically. Friedman (1970), an early economist, proposed that firms’ involvement in social affairs beyond their responsibilities and capabilities would lead to rising costs, inefficiencies and agency problems, thereby undermining corporate competitiveness. Different from it, Porter and Kramer (2002) argued that in the context of globalization, corporate philanthropy could improve the competitive environment by improving the training and education attainment and the quality of the local labor force, contributing to a win–win situation between the firm and society. Since then, the relationship between the two has sparked extensive and protracted discussions in academia. Margolis et al. (2009) conducted a meta-analysis of 167 papers and pointed out that there was a weak linear positive correlation between the two. However, this direct correlation was denied by other scholars (Surroca et al., 2010). Recently, Awaysheh et al. (2020) conducted an empirical study on the firms with the best and worst fulfillment of social responsibility in the United States industry and found that the positive correlation between the two was significant, but with endogeneity controlled, the significance disappeared, raising more questions about the existence of causality.

In addition to the above-mentioned uncorrelated and linear relationships, scholars have also proposed non-linear relationship models. Wang et al. (2008) found that Chinese firms’ corporate philanthropy has an inverted U-shaped relationship with CFP and that philanthropy can be regarded as an activity in which firms and entrepreneurs exert influence on society through the allocation of key resources. This influence, in turn, can improve performance by establishing a good brand image and getting resource feedback (p. 145). Therefore, only moderate philanthropy can achieve good results. Extra philanthropy will not only lead to rising costs but also cause serious agency problems and damage performance. Barnett and Salomon (2012) proposed the opposite positive U-shaped relationship. Their study partly adopted Friedman’s (1970) view, arguing that social responsibility is costly and will damage performance. It also pointed out that firms with higher levels of social responsibility have a stronger influence on their stakeholders and can stimulate excess returns covering costs from the latter’s feedback. Therefore, firms with higher or lower levels of social responsibility have achieved better performance than those with a moderate level of social responsibility. Yu et al. (2022) suggested that neither too much nor too little donation can improve employee performance.

Despite mixed empirical evidence, scholars have interpreted the correlation between social responsibility and performance from different theoretical perspectives.

The negative correlation holds that from the perspective of cost and benefit, as for social responsibility, we need to invest a series of resources such as personnel, finance and management, and the level of social responsibility beyond the profitability of firms will bring an additional burden to firms, making them “unable to make ends meet” (Wang et al., 2008). From the perspective of agency, the management may take social responsibility as a tool for self-interest at the cost of damaging the corporate value (Petrenko et al., 2016), leading to a “tunneling” effect; or form a protection mechanism for personal interests with a high level of social responsibility (Surroca and Tribó, 2008; Surroca et al., 2020), leading to “managerial entrenchment” effect. From the perspective of signal, social responsibility may also have a “masking effect,” that is, it becomes a tool for corporate agents to cover up operational problems. Tian and Wang (2017) tested the financial market with the PSM-DID method, and the results confirmed it – corporate social responsibility disclosure is positively correlated with the risk of a stock-price collapse. Although the specific mechanisms are different, these studies reveal that social responsibility may lead to an increase in corporate operating costs to some extent. From the perspective of stakeholders, the consequences of donations also depend on consumers’ perception of the donor’s sincerity. Tong et al. (2016) analyzed correlated data collected through a questionnaire survey and found that, in general, consumers consider local brands’ off-site donation a kind of “publicity stunt,” which may result in a backlash against the brand.

The positive correlation can also be explained from a variety of theoretical perspectives. Most studies are based on the stakeholder perspective. Wang and Qian (2011) believed that the positive impact of corporate philanthropy on performance is generated through the feedback of stakeholders. Therefore, firms with high visibility, good performance in the past and lack of political connection would benefit more from their donations. Greening and Turban (2000) found that potential employees are more willing to work for firms which actively play their role in benefiting the society. The employees’ sense of responsibility, pride and enthusiasm at work aroused in philanthropic activities can also indirectly improve the firm’s performance (Gao and Yang, 2016). Zhao and Zhang (2020) further indicated that philanthropy can enhance the citizenship behaviors of employees. Jia and Zhang (2014) found that Chinese firms with high donations can obtain higher capital market returns during the IPO stage. Based on the resource-based view, studies showed that corporate social responsibility improves the availability of scarce resources such as human resources and capital (Branco and Rodrigues, 2006), produces a reputation effect (Brammer and Millington, 2005), and can form a unique competitive advantage to promote performance improvement. In addition, from the perspective of risk management, Godfrey (2005) pointed out that corporate donations cushion the possible losses of the firm due to moral hazards in an insurance-like form.

The institutional theory is mainly used to discuss the antecedent causes of corporate social responsibility. For example, Matten and Moon (2020) and Campbell (2007), respectively, investigated the role of the institutional environment in stimulating corporate social responsibility behavior and shaping its characteristics. Chiu and Sharfman (2011) studied the positive correlation between corporate legitimacy needs and social responsibility. As far as the performance effects of social responsibility are concerned, studies have been done on the operation of multinational companies. For example, Mithani (2017) conducted a survey of the Indian market and found that the generosity of multinational corporations in the event of natural disasters can enhance their legitimacy in the host country and eliminate their disadvantages as outsiders, thus improving performance; The Chinese firms’ fulfilling social responsibility effectively cushioned the impact of low legitimacy on performance in the early stage of multinational operation (Chen and Bu, 2021). However, studies on its universality are still inadequate. Therefore, from the perspective of institutional legitimacy, this study will clarify the legitimacy basis of corporate philanthropy and its impact mechanism on business performance in the Chinese context. On this basis, we further analyze and test the moderating effect of chairman’s hometown attachment on the relationship between them. (We provide a Chinese context-based literature review in Supplementary Appendix Table 1).

## Hypothesis

### Corporate philanthropy and corporate financial performance in the Chinese context: From the perspective of institutional legitimacy

Based on the institutional theory, the survival and development of organizations need to be based on the necessary legitimacy (Meyer and Rowan, 1977). Scott (1995) pointed out that the three pillars of institutions—regulative, cognitive, and normative—provide organizations with different types of legitimacy (p. 46). Table 1 lists the emphases of the three types of legitimacy. We can see that, except that the “regulative” legitimacy comes from the expressly stipulated formal institution, the latter two categories belong to the informal institution, or implicit institutional arrangements such as people’s morality, culture and values. The latter two are similar. According to Jepperson (1991), cognitive legitimacy and normative legitimacy can be distinguished by “whether it is widely understood and accepted” and “whether it conforms to people’s morality and values.” What is widely accepted is not necessarily in line with moral judgment, such as the prevailing overtime culture of Internet firms.

**TABLE 1:** Varying emphases: Three pillars of institutions.

	Regulative	Normative	Cognitive
Basis of compliance	Expedience	Social obligation	Taken for granted
Mechanisms	Coercive	Normative	Mimetic
Logic	Instrumentality	Appropriateness	Orthodoxy
Indicators	Rules, laws, sanctions	Certification, accreditation	Prevalence, isomorphism
Basis of legitimacy	Legally sanctioned	Morally governed	Culturally supported, conceptually correct

*Source*: Scott (1995, 35p).

Firms have different degrees of legitimacy and also vary in the ability to access resources. Appropriate legitimacy can maintain the survival of firms, while extra legitimacy can improve the availability of resources to help firms develop (Zimmerman and Zeitz, 2002). From a strategic view, CSR is a typical legitimacy obtaining behavior, covering a wide range. From the legal level to the moral level and the compulsory compliance to discretion, the ability of different types of social responsibility behaviors to obtain legitimacy is not equivalent. Among them, corporate philanthropy is at the top of the pyramid model of social responsibility (Carroll, 1979). It is an entirely voluntary moral behavior with a long history in China and rich cultural implications. This study holds that corporate philanthropy in China has both normative legitimacy (moral evaluation) and cognitive legitimacy (historical inheritance and public acceptance).

First of all, good deeds have very high normative legitimacy under the enlightenment of Chinese traditional culture. In Chinese traditional culture, “do many good things” has always been encouraged by the government and praised by Confucian scholars and the people. There are many relevant teachings in the classic works of Confucian, such as “In obscurity, scholars would maintain their integrity. In times of success, they would make perfect the whole empire”^2^, and “When people of high status become wealthy, they will widely advocate moral standards, and when ordinary people become wealthy, they will behave morally according to their means”^3^, which means after being wealthy, it is selfish to please one self only but a noble behavior to help others. *The Analects of Confucius* also said that “The man of moral integrity takes morality as the first important thing” (morality should be the priority over material interests), “The whole world is one community”^4^, “The benevolent love others,” “The man of virtue, while establishing himself and pursing success, also works to establish others and enable them to succeed as well,”^5^ and “To gather talented people by sharing the wealth”^6^ … These classic teachings all convey that caring only for oneself is not advocated and that providing kindness to others is the right thing for the rich. In addition, the media and the public’s attention, encouragement, praise and supervision of corporate philanthropy not only play a role in advertising and publicity, but improve the visibility and reputation of firms, which in turn continuously strengthens the normative legitimacy of philanthropy.

Secondly, good deeds have high cognitive legitimacy in Chinese history. According to the local chronicles of Jiangsu and Zhejiang provinces, Chinese businessmen were active in philanthropy, starting from the late Ming Dynasty to the early Qing Dynasty and ending in the early Republic of China because of the war (Liang, 2001); Modern entrepreneurs’ philanthropy is also closely related to the ideas of “benevolence” and “coexistence of justice and benefit” in Confucianism (Xu et al., 2020). For a long time, philanthropy has been widely welcomed and praised by the Chinese people and also recognized and rewarded by the government. According to historical records, engaging in philanthropy has been a consistent behavior of Chinese entrepreneurs or wealthy people to meet social expectations and gain legitimacy since ancient times. A good reputation for one’s philanthropic activities can bring the donor excess cognitive legitimacy and stronger social influence.

Extra legitimacy can help gain positive feedback from stakeholders and provide development resources for firms. In the Chinese context, stakeholders do not deliberately separate entrepreneurs from their organizations, especially for private firms, the chairman of which is usually the actual controller and spokesperson of the firm. Corporate philanthropy represents the entrepreneur’s “benevolence” and “justice” and conveys the donor’s personal qualities such as responsibility and reliability, which can be transferred to the organizational image to eliminate the asymmetry of internal and external information to a certain extent, and give positive psychological implications for consumers, investors, partners, local government and other stakeholders. As a result, stakeholders imperceptibly have some favorable judgments on the corporate image and reputation, such as “entrepreneurs have higher morality, the products are more reliable; the firm is concerned about social development, pays attention to the needs of stakeholders, and has long-term planning instead of focusing on immediate interests.” Consequently, the firm welcomes more consumers (Brammer and Millington, 2005; Mithani, 2017), receives the government’s trust and support (Flammer, 2018), is favored by investors (Jia and Zhang, 2014), and has employees’ work efficiency been improved (Gao and Yang, 2016). As a direct property gift, corporate philanthropy gives a clear signal, which significantly simplifies the quantitative judgment strategy of stakeholders on the goodwill of firms. Therefore, with the increase in the donation amount, the firm’s ability to acquire legitimacy and resources also improves, which is reflected in business performance.

Hypothesis 1 *Corporate donation is positively correlated with CFP.*

### Moderating role of chairman’s hometown attachment: Pressure or resource?

Hometown attachment not only implies a close man–land relationship, but also indicates that firms that have been operating long in the region have made themselves a part of the local social network and are more legitimate than non-local firms. In terms of pressure, high legitimacy may force the firm to make high donations, thus resulting in failure to recover their costs and impairing their performance; in terms of resources, hometown attachment can enhance the emotional conformity and degree of reciprocity between the firm and local stakeholders, better leveraging the legitimacy resources acquired through philanthropic activities. Consequently, there may be two distinct moderating effects of chairman’s hometown attachment on the corporate philanthropy–performance relationship.

#### Pressure theory – Negative moderating role

According to the institutional theory, firms need to behave conforming to social cognition and expectation to obtain legitimacy. Corporate philanthropy is a typical act for this purpose. When the chairman features hometown attachment, it means that the firm has been operating in the region for a long time, so it is well-versed in local rules, culture, and customs and has been a part of the local community, and actively participates in social activities (Lewicka, 2005; Scannell and Gifford, 2010). This higher social identity gives these firms a higher legitimacy position than non-local ones (just as in the studies on international trade, the overseas operation may turn into an outsider disadvantage to multinationals (Zaheer, 1995), a result of a low legitimacy position). However, as rights match obligations, high legitimacy also implies higher stakeholder expectations, especially in terms of social responsibility (Chiu and Sharfman, 2011). That’s why local entrepreneurs are more active and responsive in philanthropic activities (Hu et al., 2017); otherwise, they are violating the high expectations of their social responsibility associated with their high legitimacy status, which also does not conform to the cultural tradition of universal responsibility and their deep affection for the hometown. For this reason, the high donation of firms with hometown attachment is likely to be taken for granted by stakeholders, thus failing to stimulate the resource feedback of the latter, which may offset the superiority in legitimacy brought by corporate philanthropy or even negatively affect the near-term performance of the firm due to the raised operation cost.

Based on the above legitimacy pressure hypothesis, this study puts forward the first competing hypothesis:

Hypothesis 2a *Ceteris paribus, the hometown attachment of the chairman of the donating firm has neither a significant effect nor a weakening effect on the positive relationship between donation and performance.*

#### Resource theory – Positive moderating effect

Because legitimacy is solidly accepted as a factor of evaluation, the acquisition of legitimacy resources is separated from the recipients’ subjective thoughts. On the one hand, people reward sincere donations while resenting hypocritical or self-interested ones. For example, Godfrey (2005) cites the case of AT&T Group in the United States, in which AT&T Group funded the pro-choice group at first but later withdrew its funding under pressure from the pro-life group. The result came as its offending both groups and a performance decline. Godfrey pointed out that AT&T’s breaking its promise made its donation, in the eyes of stakeholders, ingratiatory rather than a bona fide action, thereby resulting in its economic failure. In recent years, as social responsibility is widely recognized and undertaken, the motivation of corporate philanthropy is becoming more strategic to adapt to fierce market competition (Saiia et al., 2003), and entrepreneurs are also more likely to be motivated by self-interest (Petrenko et al., 2016). Therefore, whether the philanthropic behavior is sincere (altruistic) dramatically affects the stakeholders’ willingness to reward corporate philanthropy with resources and legitimacy. However, motivation is just a psychological state, which is not easy to be sensed by others who can merely collect some explicit information, unless the one makes a clear explanation. However, the hometown attachment characteristic provides this kind of implicit information and emphasizes the emotional conformity between the firm and local stakeholders. According to Scannell and Gifford (2010), hometown attachment conveys entrepreneurs’ recognition, acceptance, and dependence on the unique cultures, business principles, interpersonal relationships, and other social contents of the hometown, thus enhancing entrepreneurs’ willingness to contribute to the society. At the same time, in the Chinese context featuring a differential mode of association and ripple-like interpersonal relationships, it is common to be very concerned about those close and estranged with those who are not, and helping those who are distant rather than those closer would be regarded as showmanship and hypocrisy (Tong et al., 2016). On the other hand, it is easier for the stakeholders to consider donations sincere and trustworthy if such donations are out of donators’ affection for their hometown and kindness to neighbors. Otherwise, hometown attachment strengthens firms’ altruistic motivation for donations. It shortens the psychological distance between the firm and stakeholders and helps the firm fully access the legitimacy resources in the environment.

On the other hand, the stakeholder influence capacity^7^ of firms varies (Barnett, 2007). Loyal stakeholder groups are more concerned about firms’ fulfillment of social responsibilities and always give more positive feedback, so firms with loyal stakeholders can, in turn, benefit from their high-level fulfillment of social obligations. According to it, the superiority in legitimacy and social network resources obtained in the long-term operation in the local area also enhance firms’ influence on local key stakeholders. Chinese culture advocates the coexistence of justice and shared interests. Relationship is regarded as the basis and prerequisite for reciprocity. Hometown attachment is considered an essential emotional bond, the glue and catalyst for establishing a good and mutually beneficial relationship between the firm and stakeholders, and the basis of information advantage, which promotes the local key stakeholders’ understanding and recognition of the donating firm. Also, the nostalgic feelings of hometown make firms’ philanthropic behavior more humanistic, enhancing the recognition, trust, and intimacy of key stakeholders to the firm and further enhancing their willingness and intensity of resource feedback, which in turn improves firms’ influence on its stakeholders.

Therefore, from the perspective of legitimacy resources, in a donation scenario, compared with other firms, donating firms with hometown attachment can leverage more legitimacy resources from the external environment for their development by strengthening emotional connections with stakeholders and enhancing their influence on stakeholders. Fuyao Glass in Fujian Province and Tuoren Group in Henan Province are two typical successful cases. Both firms are industry leaders with influence nationwide, and both chairmen have long been engaging in philanthropy and expressed their nostalgic feelings for their hometown. Their prosocial behaviors have received positive feedback from local stakeholders, including a series of high-quality development resources, contributing to a benign relationship of coexistence and co-prosperity with the local society.

Based on the above legitimacy resources hypothesis, this study puts forward the second competing hypothesis:

Hypothesis 2b *Ceteris paribus, the hometown attachment of the chairman of the donating firm can strengthen the positive correlation between donation and performance.*

## Materials and methods

### Data and sample

#### Sample source

As of the time of data collection, there was only personal characteristic information of executives in 2018 and before in the GTA CSMAR database, with a small amount of data before 2008, the year when the Wenchuan earthquake occurred, and data of 2008 may be significantly interfered due to it. So, this study samples A-share non-financial insurance firms listed in Shanghai and Shenzhen stock exchanges from 2009 to 2018, excluding backdoor listed firms, ST, and *ST companies. Finally, a total of 19,836 firm-year observed values were obtained. Preliminary statistics show that only 536 firms have at least one donation record, with a sum of 1,724 year-firm observed values, accounting for 16.71% of the original samples. All the econometric analysis in this study is carried out with state15.1.

#### Moderator

*Hometown attachment* is the key variable in this study. Hu et al. (2017) identified this characteristic by judging whether the place of origin of the chairman and the general manager is the same as the business domicile. The method is widely adopted by relevant research. This study also defines hometown attachment if the business domicile and the chairman’s place of origin or birth^8^ are the same. Because GTA CSMAR database of executives’ characteristics only includes a small part of chairman’s native place information, we make use of search engines like Baidu and Sogou, public reports like Sino Financials, and corporate annual reports to collect data of other firms that have once donated since 2009. This study samples the chairman instead of the general manager because, in the Chinese context, the chairman is usually the supreme leader and spokesman of a firm. Hu et al. (2017) also confirm that the general manager plays an insignificant role. Our data collection method saves a lot of time. Generally, the business domicile or the chairman’s place of origin or birth is accurate to the province. When the province is the same, it is deemed that the chairman has hometown attachment, and the assigned value is 1. When the province information is inconsistent or the chairman’s personal information is not disclosed, it is deemed that the chairman has no hometown attachment, and the assigned value is 0. This is also consistent with the way external stakeholders collect and analyze information. Based on the data collected, about 37% of the donating firms feature hometown attachment (for statistics, see the Supplementary Appendix Table 2).

#### Independent variable

There are two common ways to measure the corporate *donation*: One is to measure the absolute intensity of donation with the donation amount, and the other is to measure the relative intensity with the ratio of donation amount to the asset size or operating income of the business. The amount of donation is often the first focus of consumers and the basis for the government to measure a firm’s contribution to society. Hence, this study uses the absolute intensity of donation to measure the donation, recorded as the logarithm of firms’ annual donation amount.

#### Dependent variable

The dependent variable in this study is *CFP*. There are many ways to measure CFP. Among them, the market performance by Tobin Q and the accounting performance by ROA are the most common two. However, considering that the Tobin Q theory is controversial in China, we adopt accounting performance as the main effect and market performance for the robustness check. The dependent variable of the Probit model in the first stage is the dummy variable of “whether the firm participates in donation” (*donation_if*). If the donation amount in the current year is greater than zero, the assigned value is 1; otherwise, the value is 0.

#### Control variable

Selection of control variables. Referring to research in this field, we control the variables that may affect CFP in the second-stage regression, including *age, size, debt*, and other corporate characteristics commonly used to measure CFP. Industry characteristics may affect the relationship between corporate donation and performance, so the *Herfindahl–Hirschman Index (HHI)* is controlled. Mcwilliams and Siegel (2000) argue that corporate R&D affects the relationship between social responsibility and performance, so *R&D* is also controlled. Corporate governance also impacts the relationship between donation and performance, so *board size* and *TMT compensation* are controlled. The degree of local marketization may affect CFP through the business environment. We use the marketization index released by the National Economic Research Institute (Hu et al., 2019). The index is only updated to the year 2016, and those of missing years are calculated according to the average growth rate of the marketization index over the past years. Although firm ownership is often considered a control variable that affects CFP, it is found during the empirical pre-test stage that for all the eight regression models obtained by the two regression methods and the two dependent variable measurement methods adopted in this study, the impact of firm ownership is not significant. Therefore, firm ownership is excluded for the sake of model simplicity to reduce the degree of freedom.

In the first-stage donation probability estimation model, in addition to *age, size, HHI, board size* and *TMT compensation, CEO duality, international trade* and *regional Confucianism* are also controlled. Since Confucianism has controlled the differences at the regional level, the model does not incorporate local marketization. Industry classification is subject to *The Guidelines for the Industry Classification of Listed Companies* (2012 Revision), and the industry and year fixed effects are controlled as dummy variables in the two-stage model. The exogenous variable of regional Confucianism is used as the exclusivity constraint variable. It has been proved that Confucianism strengthens the frequency and level of donation (Xu et al., 2020), but has no direct effect on performance. This variable is referred to as the ratio of the number of Confucius temples to the population, based on the measurement proposed by Kung and Ma (2014). See Table 2 for definitions of variables.

**TABLE 2:** Variable definitions.

Category	Variable	Measurement method	Data source
Dependent variable	*ROA*	Net profit/total assets at year-end	CSMAR
	*Tobin Q*	(Market value of equity + market value of net debt)/total assets at period end	CSMAR
	*Generation_if*	If the firm participates in the donation in the current year, the assigned value is 1; otherwise, it is 0	CSMAR
Independent variable	*Donation*	Ln (donation amount)	CSMAR
Moderator	*Hometown attachment (HA)*	If the chairman’s place of origin or birth is located in the same province as the business domicile, the assigned value is 1; otherwise, it is 0	Collected separately
Control variable	*Herfindahl–Hirschman Index (HHI)*	Sum of squares of percentages of operating income of all firms in each industry in the current year	CSMAR
	*R&D*	If the firm has R&D input, the assigned value is 1; otherwise, it is 0	CSMAR
	*Marketization*	Marketization index of China; data of the missing years shall be calculated according to the average growth rate of the market-oriented index over the years	National Economic Research Institute
	*Board size*	Ln (number of board members)	CSMAR
	*TMT compensation*	Ln (total compensation of the top three executives)	CSMAR
	*Debt*	Debt-to-asset ratio, winsorized at level 1%	CSMAR
	*Size*	Ln (total operating income)	CSMAR
	*Age*	Year of study – year of establishment	CSMAR
	*CEO duality*	If the Chairman is concurrently the CEO, the assigned value is 1; otherwise, it is 0	CSMAR
	*International trade*	If the firm has overseas operating income, the assigned value is 1; otherwise, it is 0	CSMAR
Exclusive constraint variable	*Confucianism*	The ratio of the number of Confucius temples in the province where the firm is located to the population at the end of the study year	Publicly available literature, National Bureau of Statistics

The number of Confucius Temples in the region is from the *Research of Global Confucius Temples* by Kong (2011) and is revised based on the number of Confucius Temples in Shanxi verified by Meng (2018); the population of each province at the end of the year is from the National Bureau of Statistics.

### Empirical model design

The initial sample of this study is unbalanced panel data. Since the firms with donation records account for less than one-fifth of the total firms, there may be serious sample selection bias that would lead to endogeneity problems. Referring to the research strategies commonly used by scholars in the field (e.g., Gao and Yang, 2016; Bose et al., 2022), we use the Heckman two-stage model for correction. The principle is to use the Probit model to estimate the probability of sample firms participating in donations and then to include the calculated and adjusted inverse Mills ratio in the second-stage regression (only for firms with donations) to eliminate the selection bias of the sample.

Since nearly one-third of the firms have only one donation for the second-stage regression, using samples as mixed cross-section data would lead to a more effective estimate. So this study uses OLS to estimate with industry and year fixed. Two methods are adopted to correct the possible heteroscedasticity of the sample. First, the natural logarithm of the data with a large standard deviation is taken or such data are measured in proportion, and then the variance is compressed. Second, the influence of heteroscedasticity is eliminated by using robust criteria.

The equation for the retest in the second step of this study is as follows:


(1) $$Performance_{i}=\beta_{0}+\beta_{1}Donatoin_{i}+\beta_{2}IMR_{i}+\beta_{3}Controls_{i}+Year+Industry+\varepsilon$$

(2) $$\;Performance_{i}=\beta_{0}+\beta_{1}Donation_{i}+\beta_{2}HA_{i}+\beta_{3}Donation_{i}$$

$$\;*HA_{i}+\beta_{4}IMR_{i}+\beta_{5}Controls_{i}$$

+Year+Industry+ε

where, *Performance*_*i*_ represents the performance of firm *i*. *DN*_*i*_ represents the natural logarithm of firm *i*’s donation. *HA*_*i*_ represents the chairman’s hometown attachment of firm *i*. *IMR*_*i*_ represents the inverse Mills ratio. *Controls*_*i*_ is the control variable, *Year* and *Industry* represent the year dummy variable and the industry dummy variable, respectively, and ε represents the error term.

## Results

### Results and analysis

Table 3 reports the statistics and correlation coefficient matrix of first-stage variables that predict firms’ probability of participating in donation. The selected control variables, except for international trade, are significantly correlated with the probability of corporate philanthropy. On average, less than 10% of non-financial A-share listed firms donated, indicating that voluntary donation was not common in China in the year of study. Table 4 reports the statistics and correlation coefficient matrix of all explanatory variables used to predict the donation in the second stage, with all correlation coefficients below 0.6 and the variance expansion factor (VIF) of each model below 10, showing no significant multicollinearity. The correlation coefficient between corporate philanthropy and accounting performance (ROA) is significantly positive (0.15), consistent with Hypothesis 1. The significant positive correlation between hometown attachment and firms’ accounting performance, market performance and donation suggests that firms with hometown attachment have relatively better performance and higher social responsibility. To eliminate multicollinearity, all variables are centralized.

**TABLE 3:** Descriptive statistics and correlation matrix of Heckman’s first-stage variables.

No.	Variables	Mean	S.D.	Min.	Max.	*1*	*2*	*3*	*4*	*5*	*6*	*7*	*8*	*9*
1	*Donation_if*	0.087	0.282	0.000	1.000									
2	*HHI*	0.104	0.107	0.015	1.000	0.056***								
3	*Age*	17.061	5.566	1.000	63.000	−0.015**	−0.087***							
4	*Size*	21.439	1.498	13.537	28.692	0.285***	0.083***	0.086***						
5	*TMT compensation*	14.257	0.712	10.361	18.049	0.153***	−0.049***	0.196***	0.415***					
6	*Board size*	2.402	0.312	1.099	3.526	0.116***	0.088***	0.139***	0.258***	0.072***				
7	*CEO duality*	0.252	0.434	0.000	1.000	−0.059***	−0.064***	−0.063***	−0.171***	0.017**	−0.214***			
8	*Confucianism*	0.010	0.005	0.002	0.032	0.019***	0.039***	−0.110***	–0.001	−0.168***	0.037***	−0.069***		
9	*International trade*	0.589	0.492	0.000	1.000	–0.011	−0.097***	−0.074***	0.079***	0.058***	−0.113***	0.086***	0.004	
10	*R&D*	0.718	0.450	0.000	1.000	−0.043***	−0.141***	–0.001	−0.047***	0.101***	−0.208***	0.140***	−0.017**	0.319***

*N* = 19,836; ***p < 0.01, **p < 0.05, *p < 0.1.

**TABLE 4:** Descriptive statistics and correlation matrix of Heckman’s second-stage variables.

No.	Variables	Mean	S.D.	Min.	Max.	*1*	*2*	*3*	*4*	*5*	*6*	*7*	*8*	*9*	*10*	*11*
1	*ROA*	0.052	0.051	–0.214	0.381											
2	*Tobin Q*	2.211	1.574	0.744	16.362	0.517***										
3	*Donation*	4.944	2.059	–1.609	14.509	0.148***	−0.064***									
4	*HA*	0.368	0.482	0.000	1.000	0.156***	0.087***	0.087***								
5	*HHI*	0.123	0.123	0.015	1.000	−0.046*	−0.070***	0.148***	−0.136***							
6	*R&D*	0.655	0.475	0.000	1.000	0.002	0.093***	−0.064***	0.120***	−0.114***						
7	*Marketization*	7.845	1.764	2.330	10.830	0.010	–0.040	0.003	−0.087***	−0.046*	0.163***					
8	*Board size*	2.519	0.313	1.609	3.526	−0.168***	−0.209***	0.069***	−0.080***	0.049**	−0.154***	−0.144***				
9	*TMT compensation*	14.610	0.736	12.571	17.406	0.143***	−0.081***	0.300***	0.028	−0.090***	0.039	0.282***	0.090***			
10	*Debt*	0.501	0.195	0.050	0.928	−0.502***	−0.501***	0.145***	−0.113***	0.056**	−0.177***	−0.068***	0.258***	0.161***		
11	*Size*	22.824	1.675	18.835	28.693	−0.115***	−0.412***	0.483***	−0.059**	0.230***	–0.0330	0.065***	0.239***	0.375***	0.541***	
12	*Age*	16.799	5.559	1.000	50.000	−0.069***	−0.095***	−0.066***	–0.012	−0.143***	0.057**	0.243***	0.145***	0.251***	0.054**	–0.026

*N* = 1,724; ***p < 0.01, **p < 0.05, *p < 0.1.

Table 5 reports the regression results from the first-stage Heckman model, which are used to estimate firms’ probability of participating in donation. Control variables, like *TMT compensation, board size, regional Confucianism, R&D*, firm *age* and firm *size*, have significant positive effects on donation probability. At the same time, *international trade* and *debt* are negatively correlated with it, and *HHI* and *CEO duality* are not significantly correlated with it. Overall, the estimated results are consistent with the existing conclusions. There is a significant correlation between regional Confucianism, an exclusive constraint variable, and donation probability, which shows its effectiveness. Based on the regression results, the inverse Mills ratio (*IMR*) is calculated and added to the subsequent regression steps to correct the sample selection bias.

**TABLE 5:** Probit estimation: Firms’ probability of participating in donation.

Variables	*Donation_if*
*HHI*	0.2345
	(0.1692)
*TMT compensation*	0.2416***
	(0.0235)
*Board size*	0.1354***
	(0.0495)
*CEO duality*	–0.0255
	(0.0380)
*Confucianism*	11.3250***
	(2.5485)
*International trade*	−0.1063***
	(0.0344)
*R&D*	0.1411***
	(0.0439)
*Age*	0.0065**
	(0.0028)
*Size*	0.3681***
	(0.0139)
*Debt*	−0.7125***
	(0.0912)
*Cons*	−13.9086***
	(0.4333)
*N*	19,836
*Pseudo R* ^2^	0.2184

***p < 0.01, **p < 0.05, *p < 0.1; robust standard errors are in parentheses; year and industry effects are controlled.

Table 6 reports the results of Heckman’s second-stage hierarchical regression. Model (1) is a baseline model with only control variables; Model (2) adds an explanatory variable *donation* to examine the relationship between donation and CFP; Model (3) adds *hometown attachment*; Model (4) adds the interaction item between hometown attachment and donation to verify whether hometown attachment has a moderating effect. The IMR of all four models is significant at 1%, indicating that the sampled firms have a strong selection effect on whether to participate in donation and that the decision can influence the CFP. The Heckman model is essential in this study. As shown in Table 6, the fitting degree of the four models constantly improves, indicating that the overall explanatory ability of the models improves with the addition of explanatory variables.

**TABLE 6:** Relationship between donation and firm performance and the moderating effect of hometown attachment.

Variables	(1)	(2)	(3)	(4)
*Donation*		0.0022***	0.0021***	0.0021***
		(0.0005)	(0.0005)	(0.0005)
*Hometown attachment*			0.0044**	0.0044**
			(0.0020)	(0.0020)
Donation* Hometown attachment				0.0024**
				(0.0011)
*HHI*	−0.0386***	−0.0383***	−0.0355***	-0.0338***
	(0.0108)	(0.0109)	(0.0111)	(0.0111)
*R&D*	0.0003	0.0006	0.0002	0.0003
	(0.0034)	(0.0034)	(0.0034)	(0.0034)
*Marketization*	–0.0008	–0.0006	–0.0006	-0.0006
	(0.0007)	(0.0007)	(0.0007)	(0.0007)
*Board size*	−0.0128***	−0.0120***	−0.0116***	-0.0118***
	(0.0035)	(0.0035)	(0.0035)	(0.0034)
*TMT compensation*	0.0077***	0.0069***	0.0071***	0.0068***
	(0.0020)	(0.0020)	(0.0020)	(0.0020)
*Debt*	−0.1451***	−0.1425***	−0.1431***	-0.1447***
	(0.0084)	(0.0085)	(0.0084)	(0.0085)
*Size*	0.0027	0.0012	0.0018	0.0026
	(0.0020)	(0.0020)	(0.0020)	(0.0021)
*Age*	–0.0001	–0.0000	–0.0001	-0.0001
	(0.0002)	(0.0002)	(0.0002)	(0.0002)
*IMR*	−0.0180**	−0.0178**	−0.0161**	-0.0147**
	(0.0071)	(0.0071)	(0.0072)	(0.0073)
*Cons*	0.0996***	0.0954***	0.0921***	0.0879***
	(0.0251)	(0.0246)	(0.0243)	(0.0241)
*N*	1,724	1,724	1,724	1,724
*R* ^2^	0.4846	0.4898	0.4912	0.4929

***p < 0.01, **p < 0.05, *p < 0.1; robust standard errors are in parentheses; year and industry effects are controlled.

**FIGURE 1:**
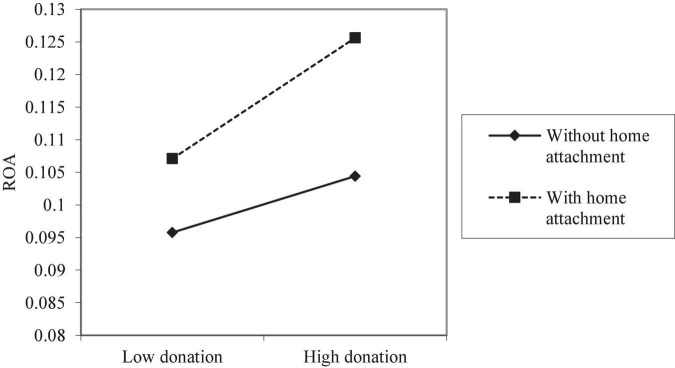
Moderating effect of hometown attachment on the donation–performance relation.

Model (2) shows a significant positive correlation between donation and CFP (β = 0.002, *p* < 0.01), supporting Hypothesis 1 (i.e., corporate philanthropy has a positive effect on CFP). The variable hometown attachment added into Model (3) is significantly positively correlated with performance (β = 0.004, *p* < 0.05), meaning the donating firm’s hometown attachment can have a positive impact on performance alone, which is also related to the advantageous, high legitimacy the firm has gain during its long-term operation in the region. Model (4) shows that the coefficient of the interaction between entrepreneurs’ hometown attachment and donation is significantly positive (β = 0.002, *p* < 0.05), which means when an entrepreneur has hometown attachment, the effect of the same amount of corporate donations on the CFP improvement is strengthened. Referring to Gao and Yang (2016), this study maps the interaction of Model (4) to better illustrate the said effects (Figure 1). According to the regression results, the average performance of firms without hometown attachment increases by 44.73% for every standard deviation increase in corporate philanthropy compared to 100.02% for firms with hometown attachment. That is, with the same donation, the performance feedback of firms with hometown attachment is around 55.29% higher than that of other firms in the year of donation. This result supports Hypothesis 2b but rejects Hypothesis 2a, suggesting that hometown attachment plays a positive role in leveraging the legitimacy resources rather than increasing the legitimacy pressure for the donating firms.

Overall, this result indicates that the more the donation is, the better performance a firm achieves and the faster the performance of a firm with hometown attachment improves. This is because the chairman’s hometown attachment can increase the public’s recognition of the altruistic motivation behind corporate philanthropy by leveraging more legitimacy in Chinese society.

### Endogenous treatment and robustness check

According to Shingal et al. (2021) and relevant studies, there are five leading causes for the endogeneity problem in management research: selection bias, variable measurement error, reverse causality, omitted variable bias, and dynamic panel deviation. In different studies, the endogeneity problems vary. This study deals with the first four possible causes of endogeneity and tests their robustness using the following methods.

(1) To avoid sample selection bias, it is retested using the Property Score-Match (PSM) method: In the scenario presented in this study, donating firms account for only a tiny portion of the sample, making selection bias the primary cause of endogeneity. However, it can be addressed by both the Heckman two-step method and the PSM method. In terms of the main effect, the Heckman two-step model is considered and the PSM method is adopted to ensure robustness. According to whether there was a donation, all firms were divided into the treatment group (with donation) and the control group (without donation). In addition, size, age, governance characteristics, local marketization, international trade, and R&D were selected as covariates through the psestimate command and correspondingly matched between the two groups to examine the effects of treatment with and without replacement. A more accurate year-by-year matching, with and without replacement, was also adopted to eliminate the influence of time. As shown in Table 7, the performance of the treatment group is significantly higher than that of the control group. Furthermore, a multiple regression model was established using the 2,085 random variables obtained through year-by-year matching to examine the moderating effect of hometown attachment. The results are shown in Table 8: only the correlation coefficient of Model (3) significantly decreases, which may result from inadequate information accuracy in measuring donations using binary variables. The above results indicate that the conclusion is still robust with the endogeneity problems being controlled by PSM.
(2) To avoid measurement bias, dependent variables are replaced: The Tobin Q ratio of market performance instead of accounting performance is regressed and the Heckman two-step method is used. The results show that the significance and sign of the main effect are highly consistent with the preceding part (Table 9). Since the IMR in the model is not significant, the re-regression results without it are still the same and will not be reported separately.
(3) Re-check with a new model. In the Heckman’s second-stage estimation, according to the heteroscedasticity of the sample, this study uses the Feasible Generalized Least Squares (FGLS) method to re-regress the two measurement methods of CFP respectively, and reports the results in Table 10. All the four models are significant at 1%, suggesting the effectiveness of the model settings. The results are highly consistent with the previous results in terms of the sign and significance of both main explanatory variables and control variables, demonstrating the robustness of the conclusions.
(4) The explained variables are re-regressed one period in advance. The accounting and market performances in period *t* + 1 are re-regressed on the explanatory variables in period *t*. Table 11 reports the test results of OLS and FGLS. It can be seen that, despite a reduced sample size, seven of the eight models are consistent with the previous conclusions, with only Model (16) losing significance but having the same sign. This indicates that the donation of this period still has an effect on the performance of the next period, further eliminating the causal inversion problem of the model.
(5) Other robustness checks. In this study, the FGLS method is also employed for multiple regression directly on the samples participating in donation; and control variables such as CEO duality, HHI, and the proportion of independent directors are added to solve the problem of omitted control variables. The results are consistent with the previous part and will not be reported separately.

**TABLE 7:** PSM treatment effect of donation_if on outcome variable ROA.

Category	Nearest neighbor matching		Year-by-year matching	
	Without replacement	With replacement	With replacement	Without replacement
Average treatment effect	0.0178***	0.0178***	0.0083***	0.0083***
	(0.0020)	(0.0020)	(0.0020)	(0.0020)
*N*	19,836	19,836	19,836	19,836

***p < 0.01, **p < 0.05, *p < 0.1.

**TABLE 8:** Regression result of PSM treated samples.

Variables	(1)	(2)	(3)	(4)
*Donation_if*	0.0079**	0.0041	0.0047	0.0013
	(0.0032)	(0.0038)	(0.0033)	(0.0039)
Donation_if * Hometown attachment		0.0128*		0.0116*
		(0.0067)		(0.0066)
*Hometown attachment*	0.0060**	−0.0046	0.0045*	−0.0050
	(0.0027)	(0.0061)	(0.0027)	(0.0061)
*Size*	0.0066***	0.0065***	0.0071***	0.0070***
	(0.0011)	(0.0011)	(0.0011)	(0.0011)
*Board size*	−0.0114***	−0.0113**	−0.0134***	−0.0132***
	(0.0044)	(0.0044)	(0.0044)	(0.0044)
*Debt*	−0.1638***	−0.1631***	−0.1641***	−0.1635***
	(0.0088)	(0.0088)	(0.0088)	(0.0088)
*Marketization*	−0.0031***	−0.0032***	−0.0023***	−0.0024***
	(0.0008)	(0.0008)	(0.0007)	(0.0008)
*TMT compensation*	0.0140***	0.0142***	0.0141***	0.0142***
	(0.0020)	(0.0020)	(0.0020)	(0.0020)
*Age*	−0.0004*	−0.0004*	0.0001	0.0001
	(0.0002)	(0.0002)	(0.0004)	(0.0003)
*R&D*	−0.0048	−0.0051	0.0004	0.0001
	(0.0035)	(0.0035)	(0.0037)	(0.0037)
*Cons*	−0.1482***	−0.1469***	−0.1501***	−0.1487***
	(0.0321)	(0.0320)	(0.0327)	(0.0328)
*Industry effect*	Y	Y	Y	Y
*Year effect*	N	N	Y	Y
*N*	2,085	2,085	2,085	2,085
*R* ^2^	0.3253	0.3265	0.3368	0.3378

The missing variable “hometown attachment” in this sample has been added manually; ***p < 0.01, **p < 0.05, *p < 0.1; robust standard errors are in parentheses.

**TABLE 9:** Regression result of using Tobin Q as the explained variable.

Variables	(5)	(6)	(7)
*Donation*	0.0451***	0.0481***	0.0474***
	(0.0145)	(0.0146)	(0.0146)
*Hometown attachment*		−0.0895	−0.0890
		(0.0593)	(0.0592)
Donation* Hometown attachment			0.0538*
			(0.0275)
*Cons*	2.2806***	2.3485***	2.2535***
	(0.5857)	(0.5961)	(0.5861)
*Control variables*	Y	Y	Y
*N*	1,724	1,724	1,724
*R* ^2^	0.5216	0.5223	0.5234

***p < 0.01, **p < 0.05, *p < 0.1; robust standard errors are in parentheses; year and industry effects are controlled.

**TABLE 10:** Regression result of FGLS model.

Variables	*ROA*		*Tobin Q*	
	(8)	(9)	(10)	(11)
*Donation*	0.0016***	0.0017***	0.0330***	0.0362***
	(0.0002)	(0.0002)	(0.0054)	(0.0059)
*Hometown attachment*		0.0034***		−0.1231***
		(0.0009)		(0.0215)
Donation * Hometown attachment		0.0024***		0.0322***
		(0.0005)		(0.0112)
*IMR*	−0.0186***	−0.0155***	−0.1747**	−0.2000**
	(0.0023)	(0.0024)	(0.0806)	(0.0801)
*Control variables*	Y	Y	Y	Y
*N*	1,724	1,724	1,724	1,724
*Firms*	536	536	536	536

***p < 0.01, **p < 0.05, *p < 0.1; year and industry effects are controlled.

**TABLE 11:** Regression result of using T + 1 explanatory variables.

Variables	*ROA*				*Tobin Q*			
	OLS		FGLS		OLS		FGLS	
	(12)	(13)	(14)	(15)	(16)	(17)	(18)	(19)
*Donation*	0.0019**	0.0017*	0.0016***	0.0019***	0.0300	0.0309	0.0309***	0.0357***
	(0.0009)	(0.0009)	(0.0003)	(0.0003)	(0.0257)	(0.0254)	(0.0065)	(0.0078)
*Hometown attachment*		0.0012		0.0011		−0.1102		−0.1177***
		(0.0030)		(0.0013)		(0.0758)		(0.0240)
Donation * Hometown attachment		0.0026*		0.0016***		0.0598*		0.0447***
		(0.0015)		(0.0005)		(0.0350)		(0.0120)
*IMR*	−0.0235**	−0.0212**	−0.0206***	−0.0197***	0.3547	0.3502	0.1636	0.1635
	(0.0091)	(0.0092)	(0.0039)	(0.0042)	(0.3632)	(0.3693)	(0.1100)	(0.1136)
*Control variables*	Y	Y	Y	Y	Y	Y	Y	Y
*N*	903	903	903	903	903	903	903	903
*R* ^2^	0.4794	0.4814	\	\	0.5783	0.5804	\	\

***p < 0.01, **p < 0.05, *p < 0.1; year and industry effects are controlled.

## Further testing

Based on the above argument, hometown attachment mainly enhances the positive effect of corporate donation on corporate performance by leveraging more legitimacy resources, and these resources in the business environment are largely from external key stakeholders. Based on this, this study selects two key groups, government, and consumer, to investigate their response to the donation of firms with hometown attachment and verify the mechanism.

### Government response

The government provides resource support for developing firms in the region by formulating policies and providing investment opportunities and financing channels. Among them, various forms of subsidies that conform to relevant national policies are directly allocable financial resources. Sufficient subsidies can even directly bolster the performance of some firms. However, the subsidy amount is always caped. Also, it is difficult for the government to know clearly the profitability, technological innovation, taxable capacity, and employment-providing ability of each firm. Such an information asymmetry makes decisions on subsidy allocation challenging. In that case, the high legitimacy of firms turns out to be an advantage. That is, the government tends to give priority to the firms with good relations (Yu et al., 2010). At the same time, corporate philanthropy can objectively support the government in improving the local’s livelihood, acting as a bridge to a good government-enterprise relationship (Wang and Qian, 2011). Therefore, when hometown attachment leverages the legitimacy resources of donating firms, the government may provide more subsidies to these firms in the year of donation.

### Consumer response

Consumers can support or resist a firm through their purchasing decisions. Numerous studies in consumer behavior have pointed out that socially responsible firms can stimulate consumers’ purchase intentions and enhance consumers’ positive perception of product quality (Brammer and Millington, 2005; Mithani, 2017). As mentioned above, the chairman’s hometown attachment leveraged all the legitimacy resources consumers could provide, demonstrated by higher consumer trust, evaluation and active purchasing. Hence, by analyzing the change in sales ratios in the year when donations are made, we can indirectly learn whether corporate philanthropy influenced by hometown attachment enhances the attraction of the firm to consumers. That is, under the same circumstances, the sales efficiency of a donating firm with hometown attachment should be higher than those without hometown attachment.

### Regression results

In this study, government subsidy refers to government response, measured as the logarithm of the annual subsidy amount received by the firm + 1, and sales ratio (the ratio of annual sales to annual operating income) is chosen to refer to consumer response (i.e., the smaller the data is, the better the consumer response is). The explanatory variables are regressed separately by government subsidy and sales ratio, with all other control variables remaining consistent with the main effect. Since both explained variables have some values “0” (albeit in a small proportion), the Tobit regression model suitable for truncated data is adopted to ensure robustness. As can be seen from Table 12, the interaction term between donation and hometown attachment is significantly positive in both Model (1) and Model (2) and significantly negative in both Model (3) and Model (4). This means the two jointly boost the government subsidies and firms’ sales ratio, consistent with expectations. The theoretical mechanism proposed above is verified.

**TABLE 12:** Effect of hometown attachment on external key stakeholders.

Variables	*Government subsidy*		*Sales ratio*	
	OLS	Tobit	OLS	Tobit
	(1)	(2)	(3)	(4)
*Donation*	−0.0336	−0.0334	0.0019**	0.0020**
	(0.0434)	(0.0489)	(0.0009)	(0.0008)
*Hometown attachment*	0.2604	0.2679	−0.0008	−0.0011
	(0.1713)	(0.1822)	(0.0032)	(0.0029)
Donation * Hometown attachment	0.1852**	0.1925**	−0.0025*	−0.0026*
	(0.0822)	(0.0875)	(0.0015)	(0.0014)
*IMR*	2.2247***	2.2821***	0.0529***	0.0570***
	(0.7614)	(0.5744)	(0.0082)	(0.0093)
*Control variables*	Y	Y	Y	Y
*N*	1,724	1,724	1,714	1,714
*R* ^2^	0.2975		0.5479	
*Left-censored*		66		29

***p < 0.01, **p < 0.05, *p < 0.1; robust standard errors are in parentheses; year and industry effects are controlled.

## Discussion

### Theoretical implications

This study provides several theoretical implications. First, based on Scott’s (1995) theory of the three pillars of institutions, this study analyzes the legitimacy sources of corporate philanthropy and the resource transformation mechanism in the Chinese cultural context, which has particular theoretical and practical significance. From the theoretical point of view, although scholars have recognized the role of social responsibility behaviors in enhancing corporate legitimacy, they have not yet thoroughly analyzed the legitimacy sources of corporate philanthropy in different contexts and their impact mechanism on performance. This study points out that legitimacy is rooted in a specific institutional environment, coexisting with cultural concepts and promoting each other. In the Chinese context, the informal institutional environment shaped by normative and cognitive institutional pillars serves as the seedbed for corporate social responsibility behaviors to gain extra legitimacy. In this context, whether or not to identify and grasp the opportunity to obtain extra legitimacy depends not only on the entrepreneurs’ marketing concept and business ability, but also on their individual morality and cognition. In addition, this research also shows that stakeholders are the providers of legitimacy resources, so ensuring that stakeholders have sufficient resources and can reward firms’ prosocial behavior based on their independent choices is not only an essential prerequisite for a benign interaction between firms and their external environment, but also the cornerstone for the exertion of tertiary distribution and the realization of shared prosperity.

Second, this study combines environmental psychology concepts, upper echelons theory, and institutional theory to deepen the understanding of the social role of the psychological phenomenon of entrepreneurs’ hometown attachment and promote the study and application of the psychological characteristics of key executives in the field of management. Place attachment is a common psychological phenomenon worldwide, while hometown attachment is particularly common and typical in the Chinese cultural context. This study inherits and extends the existing research results (Hu et al., 2017), and verifies and supplements the positive moderating effect of hometown attachment in the context of donation. Hometown attachment as one of the psychological characteristics of executives is an important research branch on the antecedent causes of corporate social responsibility, and the focus of the upper echelons theory. Although related studies have focused on the association of CEO psychology with CSR engagement from different perspectives, such as Narcissism and cognitive style, and the influence of psychological factors embedded in life experiences, gender, and cultural background (Du et al., 2014; Petrenko et al., 2016; Galbreath, 2018; Xu and Ma, 2021; Zhao et al., 2022), the mechanism how hometown attachment affects the performance of corporate donation is under-revealed. In a foundation of upper echelons theory, Hambrick and Mason (1984) suggested that “executive characteristics–corporate strategy–corporate financial performance” is a continuous process, and Grewatsch and Kleindienst (2017) also called for more attention to the moderating role of individuals in the CSR–performance relationship. This study responds to this appeal by exploring the psychological characteristics of the chairman’s hometown attachment and also complements the research on corporate strategy–performance relationships based on upper echelons theory.

### Practical implications

This study also provides some inspirations for practice in terms of entrepreneurs’ self-cultivation and philanthropic behaviors. First, the Chinese excellent traditional culture represented by Confucianism has shaped an informal system environment where those who benefit others also benefit themselves. Among the three pillars of institutional legitimacy, regulative legitimacy is necessary for the existence of firms. In contrast, the other two (cognitive legitimacy and normative legitimacy) are the primary sources of extra legitimacy, which can be transformed into the development resources of firms. Spontaneous performance of social responsibility allows for these resources. However, such acts are often the externalization of entrepreneurs’ cognition and morality. In this regard, identifying and grasping an opportunity to obtain extra legitimacy depends not only on entrepreneurs’ marketing concept and business ability but also on their individual morality and cognition.

Second, even though entrepreneurs cannot choose their origins, in the information era, competition among firms is not merely in products and services but also in effective communication, management, and appropriate strategies on the basis of understanding the stakeholders. To improve the recognition of stakeholders, firms may properly emphasize the decision-maker’s hometown attachment when promoting in a donation scenario. Firms without this feature can focus on promoting the corporate vision, culture, behavioral consistency, and other characteristics at the organizational level.

Third, the research shows that the firms mainly obtain legitimacy resources from key stakeholders such as the government and consumers. It means the total resources these stakeholders hold can significantly affect firms’ returns from their prosocial behaviors. Therefore, it is an important prerequisite for positive interaction between a firm and its external environment to ensure that stakeholders have sufficient resources and can reward the firm’s prosocial behaviors based on their independent choices. It is also the cornerstone for the exertion of tertiary distribution and the realization of shared prosperity.

Finally, both hometown attachment and Confucianism belong to the informal system. Xu et al. (2020) pointed out that Confucianism can actively promote entrepreneurs to engage in philanthropy. This study has made further progress and points out that Confucianism also promotes firms to acquire extra legitimacy through philanthropy, and hometown attachment as one of the characteristics of traditional cultural values can further help entrepreneurs who devote themselves to philanthropy to leverage the resources brought by extra legitimacy, thereby rewarding their good deeds, enabling a virtuous cycle.

Thus, we address the practical advices in three bullets below:

• Chinese traditional culture helps to improve the altruistic motivation of enterprise managers, so we encourage managers to critically learn and inherit traditional culture.
• When a donation enterprise has a local chairman, increasing the publicity of his or her hometown attachment will benefit the donation performance of the enterprise.
• The government should foster a good formal and informal institutional environment to ensure that enterprises, consumers and other stakeholders have enough resources to encourage altruistic charitable donations, thereby enabling a virtuous cycle.

### Limitations and prospects

It should be clarified that this conclusion based on the research design of this study does not apply to particular scenarios of major national disaster emergencies. Tong et al. (2016) have demonstrated that firms’ cross-regional donations in a national natural disaster often give rise to greater reputation and legitimacy, which is due to the great social concern about the event. Due to limitations, the author can neither collect the personal information of all the executives, nor get to know whether entrepreneurs’ hometown attachment has a positive impact on philanthropy decisions, whether it mutually reinforces or counteracts the effects of other cultural features, and whether different characteristics of executive team members have synergistic or contradictory results. Future studies can be conducted regarding these questions. Also, given that transnational firms are facing unprecedented challenges due to the tense international situation and rising nationalism globally, future research can focus on the role of hometown attachment in international competition, and study how it triggers transnational firms’ actions to fulfill their social responsibilities and whether it will change the value standard of stakeholders in different positions and the reciprocal relationship between them and firms.

The results of this study also warn us that since philanthropy is rooted in a specific institutional situation, provides firms with extra legitimacy and creates value, it will also change as a result of institutional changes—-if groups no longer recognize legitimacy as an embodiment of entrepreneurs’ morality, but their duties, the positive effect of philanthropy on firms is likely to weaken or even disappear. In a word, whether to donate or not and how much to donate is an independent decision of each entrepreneur, and only on a voluntary basis can it lead to the possibility of extra legitimacy. As the Tao Te Ching says, “When people see some things as beautiful, other things become ugly. When people see some things as good, other things become bad.” Long is long only when there is something short, and high is high only when there is something low. Only when altruistic behavior is seen as an externalized manifestation of a precious inner quality and valued can it lead to self-interested outcomes.

## Conclusion

Samples in this study are data of donation and chairman’s native place of listed firms engaging in donation from 2009 to 2018. The conclusions drawn from empirical tests include: First, corporate philanthropy in the Chinese context is rich in connotations. Through corporate philanthropy, a firm can gain extra legitimacy in return and stimulate the resource feedback of stakeholders. The higher amount of corporate donations, the better performance of the firm. Second, the chairman’s hometown attachment conveys emotional conformity and improves the degree of reciprocity between the firm and its stakeholders, thereby maximizing the legitimacy resources acquired and stored through philanthropy. That’s why when the chairman of a donating firm has hometown attachment, the positive correlation between the firm’s donation and performance is more significant. Further analysis shows that the government and consumers support the firms with hometown attachment by providing them higher subsidies and purchasing from these firms.

## Data availability statement

The original contributions presented in this study are included in the article/Supplementary material, further inquiries can be directed to the corresponding author.

## Author contributions

XB designed the study, collected and analyzed the data, and wrote the manuscript. LC gave guidance throughout the whole research process and reviewed the manuscript. Both authors contributed to the article and approved the submitted version.

## Footnotes

1 See http://www.jiemian.com/article/6414339.html

2 See *Mencius*.

3 See *Records of the Historian⋅Biographies of Extremely Wealthy Businessmen*, Sima Qian, from the Western Han Dynasty.

4 See *the Book of Rites⋅the Common Wealth of Great Unity*.

5 See *the Analects of Confucius*.

6 See *the Book of Rites⋅the Great Learning*.

7 Barnett (2007, p. 803) defines it as, “the ability of a firm to identify, act on, and profit from opportunities to improve steady relationships through CSR”.

8 About 10% of the data do not indicate whether it is the place of origin or birth, so the data are combined.
