# Supplementary material for: Enhance or inhibit? Unveiling the influence of chairman’s hometown attachment on the corporate philanthropy–Corporate financial performance relationship
Source: Front Psychol. 2022 Nov 9;13:956689. doi: 10.3389/fpsyg.2022.956689 (PMC9682063; doi:10.3389/fpsyg.2022.956689)
Supplement: Supplementary file 1 [file Data_Sheet_1.pdf]

APPENDIX

Table A1: Literature review of the CSR/CP-corporate performance relationship based on Chinese evidence

Relationship	Research	Main findings
Positive	Wang and Qian (2011)	Firms with high visibility, good performance in the past and lack of political connection would benefit more from their donations.
	Jia and Zhang (2014)	Chinese firms with high donations can obtain higher capital market returns at the IPO stage.
	Khan et al.(2022)	The silent charitable donations made by Chinese non-SOEs significantly improved their performance, and this relationship is oppositely moderated by firm size and CEO salary.
	Gao and Yang (2016); Zhao and Zhang (2020)	There is a positive relationship between employees' outcomes and corporate philanthropy.
Negative	Tong et al. (2016)	Consumers consider local brands' off-site donations a kind of "publicity stunt", which may result in a backlash against the brand and harm its financial performance.
	Tian and Wang (2017)	They empirically tested the Chinese financial market with the PSM-DID method, and the results confirmed that corporate social responsibility disclosure is positively correlated with the risk of a stock-price collapse.
Non-linear shape	Chen and Bu (2021)	There is a U-shaped correlation between the CSR of internationalized Chinese firms and their CFP.
	Yu et al. (2022)	Neither too much nor too little donation can improve employee performance.

Appendix table 2: Geographic distribution of donating firms and the hometown attachment of chairmen

Geographic location of sampled firms	Total observed value	Including: observed value of those with hometown attachment	Proportion of observed value of those with hometown attachment
Shanghai	156	34	0.22
Yunnan	57	20	0.35
Inner Mongolia	8	1	0.13
Beijing	263	28	0.11
Jilin	13	4	0.31
Sichuan	58	26	0.45
Tianjin	31	2	0.06
Ningxia	5	0	0.00
Anhui	48	25	0.52
Shandong	87	56	0.64
Shanxi	30	23	0.77
Guangdong	241	69	0.29

Guangxi	11	7	0.64
Xinjiang	30	8	0.27
Jiangsu	93	31	0.33
Jiangxi	27	15	0.56
Hebei	30	12	0.40
Henan	81	37	0.46
Zhejiang	149	93	0.62
Hainan	9	0	0.00
Hubei	33	24	0.73
Hunan	32	18	0.56
Gansu	4	2	0.50
Fujian	140	72	0.51
Guizhou	7	0	0.00
Liaoning	33	22	0.67
Chongqing	11	1	0.09
Shaanxi	12	2	0.17
Qinghai	16	0	0.00
Heilongjiang	9	2	0.22
Total	1,724	634	0.37

Note: There are 1,724 observed values of sampled firms that participated in donations from 2009 to 2018. The geographic location is based on the province where the firm is registered.
